# Neuroprotective and immunomodulatory effects of superoxide dismutase on SH-SY5Y neuroblastoma cells and RAW264.7 macrophages

**DOI:** 10.1371/journal.pone.0303136

**Published:** 2024-05-14

**Authors:** Moon-Beom Kim, Su-Min Park, Ga-Hyun Lim, Yong-Hun Oh, Kyung-Won Seo, Hwa-Young Youn

**Affiliations:** Department of Veterinary Clinical Sciences, Laboratory of Veterinary Internal Medicine, College of Veterinary Medicine, Seoul National University, Seoul, Republic of Korea; Universiti Malaya, MALAYSIA

## Abstract

Superoxide dismutase (SOD) is an antioxidant enzyme that protects the body from free radicals. It has both antioxidant and immunomodulatory properties, inducing macrophage polarization from M1 to M2. Macrophages, key mediators of the innate immune response, are divided into the M1 (pro-inflammatory) and M2 (anti-inflammatory) subtypes. In this study, we aimed to assess the antioxidant and neuroprotective effects of SOD on nerve cells and its immunomodulatory effects on macrophages. We observed that SOD inhibited the accumulation of reactive oxygen species and enhanced the viability of H_2_O_2_-treated nerve cells. Furthermore, SOD reduced the degree of necrosis in nerve cells treated with the conditioned medium from macrophages, which induced inflammation. In addition, SOD promoted the M1 to M2 transition of macrophages. Our findings suggest that SOD protects nerve cells and regulates immune responses.

## Introduction

Superoxide dismutase (SOD) is present in all aerobic living cells [1]. It is generated during many cellular processes as a product of normal respiration and oxidative burst in immune cells [2]. Notably, it is regarded as the most essential line of enzymatic antioxidant defense against reactive oxygen species (ROS). Three isoforms of SOD have been identified [3,4]. SOD1 or Cu/Zn-SOD is found in intracellular cytoplasmic spaces. SOD2 and MnSOD are observed in the mitochondrial matrix. SOD3 or extracellular-SOD exists as a tetramer containing copper and zinc and is synthesized with a signal peptide directing the enzyme to the extracellular area [5,6]. It converts superoxide (O_2_^-^) radicals into hydrogen peroxide (H_2_O_2_) and ordinary molecular oxygen (O_2_) [7]. Superoxide radicals are the most important ROS involved in the progression of several inflammatory diseases [8]. The intracellular ROS concentration is controlled by antioxidant systems; SOD, catalase, and glutathione peroxidase are the main antioxidant enzymes in mammalian cells [9]. An increase in ROS concentration leads to dilated cardiomyopathy, severe anemia, neurodegeneration, and a short lifespan in an SOD knockout (KO) mouse model [10].

In addition to being an antioxidant enzyme, SOD exerts immunomodulatory effects and polarizes macrophages to the M2 phase [11]. Macrophages play important roles in the interactions between innate and adaptive immune responses [12]. They are distinctly subdivided into classical M1 and M2 categories [13]. Typically, interferon-γ or lipopolysaccharide (LPS) activate the M1 (classically activated) macrophages to produce pro-inflammatory cytokines, phagocytose microorganisms, and initiate immune reactions. M2 (alternatively activated) macrophages are activated by cytokines, such as interleukin (IL)-4, IL-10, and IL-13. Macrophages are also involved in tissue repair and wound healing [14,15]. SOD affects macrophages, and SOD KO mice exhibit diminished phagocytosis and bactericidal abilities, indicating that SOD increases bacterial clearance and reduces inflammation [16]. Additionally, SOD polarizes macrophages from the M1 to M2 phenotype, and SOD-mediated H_2_O_2_ regulates M2 gene expression at the transcriptional level via redox modulation of a key cysteine in signal transducer and activator of transcription-6 [17].

Several studies have investigated the relationship between neurodegenerative and neuroinflammatory diseases and macrophages [18,19]. Immune cells, particularly macrophages, are involved in the initiation, progression, and resolution of neuroinflammation. Cell populations are the key participants in central nervous system pathologies, including autoimmune disorders (such as multiple sclerosis) and degenerative diseases (such as amyotrophic lateral sclerosis [ALS] and Alzheimer’s disease) [20]. Therefore, SOD has attracted considerable attention for its therapeutic potential and warrants further investigation.

In this study, we aimed to investigate the antioxidant and neuroprotective effects of SOD on the human neuroblastoma cell line SH-SY5Y and its immunomodulatory effects on the LPS-treated macrophage cell line RAW264.7.

## Materials and methods

### Cell culture

Human neuroblastoma (SH-SY5Y; Korea Cell Line Bank, Seoul, Korea) and mouse macrophage (RAW264.7; Korea Cell Line Bank) cell lines were cultured in high glucose-Dulbecco’s modified Eagle’s medium (Welgene, Gyeongsan, Korea) supplemented with 100 units/mL penicillin G (Sigma-Aldrich, St. Louis, MO, USA), 100 μg/mL streptomycin (Sigma-Aldrich), and 10% fetal bovine serum (Gibco, Waltham, MA, USA) and incubated at 37 °C in a humidified atmosphere containing 5% CO_2_. The culture medium was replaced every 2–3 days. The cells were subcultured after they reached 70–80% confluency.

### Cell viability test

Cell viability was assessed by the cell counting kit-8 (CCK-8) assay (D-Plus CCK Cell Viability Assay Kit; Dong-in Biotech, Seoul, Korea) to determine whether SOD had any influence on the growth of SH-SY5Y neurons and RAW264.7 cells. SH-SY5Y cells were seeded at a density of 2.0 × 10^4^ cells/well, whereas RAW264.7 cells were seeded at a density of 1.0 × 10^4^ cells/well in 96-well plates, followed by manufacturer’s instructions. Thereafter, various concentrations of SOD were added (0, 0.01, 0.1, 1, 10, 100, 200, 500, and 1000 μM for SH-SY5Y cells; 0, 10, 100, 200, 500, and 1000 μM for RAW264.7 cells) to the wells, and cytotoxic effects were evaluated using the CCK-8 assay.

### ROS detection test

SH-SY5Y cells were treated with 1 μM SOD for 24 h, and H_2_O_2_ was added at 200 μM. Intracellular ROS levels were measured based on the detection of the fluorescent product, 2′,7′-dichlorofluorescein, obtained from the oxidation of 2′,7′-dichlorodihydrofluorescein diacetate (H_2_DCFDA). Cells were incubated in a medium containing 10 μM H_2_DCFDA for 1 h. After incubation, the medium was removed, and the cells were washed with Dulbecco’s phosphate-buffered saline (D-PBS). The stained cultures were analyzed for green fluorescence using an inverted fluorescence microscope (EVOS FL microscope; Life Technologies, Darmstadt, Germany) and a fluorescence microplate reader (Infinite M200 Pro; TECAN, Zurich, Switzerland).

### Immunofluorescence (IF) analysis

RAW264.7 macrophages were fixed with 4% paraformaldehyde for IF staining and blocked with a buffer containing 5% bovine serum albumin (Sigma-Aldrich) and 0.1% Triton X-100 (Sigma-Aldrich) for 30 min. Cells were then incubated with antibodies against allophycocyanin-conjugated CD11c (1:100; Santa Cruz Biotechnology) and fluorescein isothiocyanate (FITC)-conjugated CD206 (1:100; Santa Cruz Biotechnology) at 4 °C for 1 h. After three washes, cells were mounted with the Vectashield mounting medium containing 4′,6-diamidino-2-phenylindole (Vector Laboratories, Burlingame, CA) and observed under an EVOS FL microscope (Life Technologies).

### RNA extraction, cDNA synthesis, and real-time polymerase chain reaction (PCR)

Total RNA was extracted from RAW264.7 cells using the Easy-Blue RNA extraction kit (iNtRON Biotechnology, Seongnam, Korea) according to the manufacturer’s instructions. CellScript All-in-One 5X First Strand cDNA Synthesis Master Mix (Cell Safe, Yongin, Korea) was used to synthesize cDNA, and RNA expression was analyzed using 400 nM forward and reverse primers (Bionics, Seoul, Korea) and AMPIGENE qPCR Green Mix Hi-ROX with SYBR Green dye (Enzo Life Sciences, Farmingdale, NY, USA) on an Applied Biosystems QuantStudio 5 qPCR System (Thermo Fisher Scientific, Waltham, MA, USA). The expression of each gene was normalized to that of glyceraldehyde 3-phosphate dehydrogenase (GAPDH). All primer sequences are listed in Table 1.

**Table 1:** Sequences of polymerase chain reaction (PCR) primers used in this study.

Species	Gene	Sequence
Mouse	*IL-6*	F: AGTTGCCTTCTTGGGACTGA
		R: TCCACGATTTCCCAGAGAAC
	*IL-1β*	F: TGGACCTTCCAGGATGAGGACA
		R: GTTCATCTCGGAGCCTGTAGTG
	*TNF-α*	F: CCCTCACACTCAGATCATCTTCT
		R: GCTACGACGTGGGCTACAG
	*GAPDH*	F: AGTATGTCGTGGAGTCTACTGGTGT
		R: AGTGAGTTGTCATATTTCTCGTGGT

### Conditioned medium for RAW264.7 cell culture

To induce M1 macrophage polarization, 200 ng/mL LPS was added to the RAW264.7 cell culture. After 6 h, the cells were washed with PBS to ensure that the cytokines used in the culture were completely removed, and the medium was replaced with the complete culture medium. After culturing for another 24 h, the medium supernatant was collected, centrifuged at 1200 rpm for 5 min, and filtered through a 0.22 μm-pore filter to remove the debris.

### Treatment of SH-SY5Y cells with the conditioned medium

SH-SY5Y cells were seeded in a 6-well cell-culture plate at a density of 1.0 × 10^6^ cells/well. After cell adhesion, the medium was replaced with a fresh medium consisting of 30% conditioned medium and 70% original medium. To compare the neuroprotective effects of SOD, some groups were treated with 1 U/mL SOD. All plates were incubated at 37 °C for 24 h.

### Annexin V staining

SH-S5Y5 cells treated with the conditioned medium and SOD were stained with annexin V-FITC/propidium iodide (PI) (BD Biosciences, San Jose, CA) and subsequently analyzed using a fluorescence-activated cell sorting instrument (FACS Aria II; BD Biosciences), according to the manufacturer’s instructions. Stained cells were then categorized as follows: PI- and annexin V-negative (lower left quadrant; normal), PI-negative and annexin V-positive (lower right quadrant; early apoptotic), PI- and annexin V-positive (upper right quadrant; late apoptotic), and PI-positive and annexin V-negative (upper left quadrant; necrotic).

### Statistical analyses

Statistical analyses were performed using GraphPad Prism (version 6.01) software (GraphPad Software, La Jolla, CA, USA). Data were analyzed using Student’s *t*-test and one-way analysis of variance, followed by Bonferroni’s multiple comparison test. Data are represented as the mean ± standard deviation. Differences were considered statistically significant at *P* < 0.05.

## Results

### Viability test of SH-SY5Y and RAW264.7 cells using SOD

**Fig 1:**
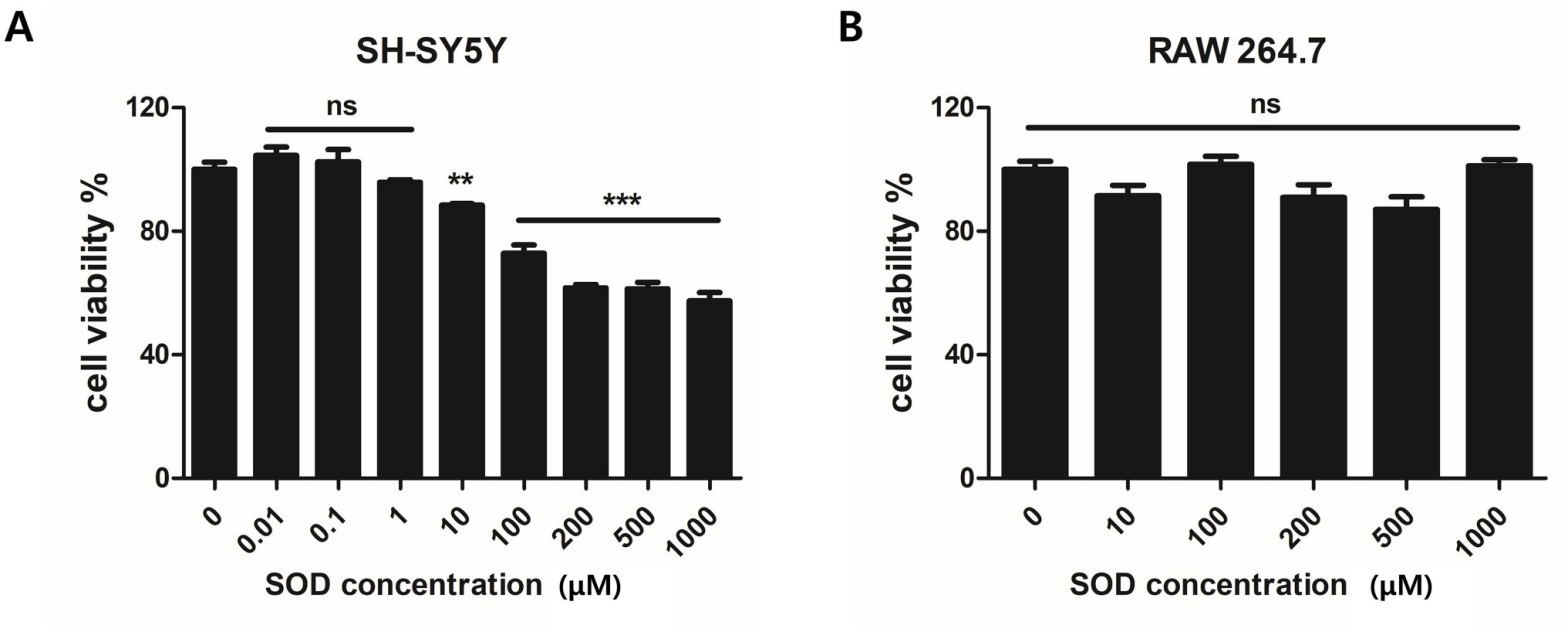
Viability assessment of SH-SY5Y neuronal cells and RAW264.7 macrophages using the cell counting kit (CCK)-8 assay. (A) In SH-SY5Y neuronal cells, viability was reduced by superoxide dismutase (SOD) concentrations > 10 mM. (B) In RAW264.7 macrophages, viability was not affected by up to 1000 mM SOD. **P < 0.01 and ***P < 0.001. ns, not significant.

The cytotoxicity of SOD was evaluated using the CCK-8 assay. Cell viability was determined after treating SH-SY5Y and RAW264.7 cells with various concentrations of SOD. In SH-SY5Y cells, viability was stable with 0, 0.01, 0.1, and 1 μM of SOD but decreased at concentrations > 10 μM (*P* < 0.01 and *P* < 0.001; Fig 1A). In contrast, the viability of RAW264.7 cells was not affected by SOD up to 1000 μM (Fig 1B).

### Neuroprotective effects of SOD on SH-SY5Y cells

**Fig 2:**
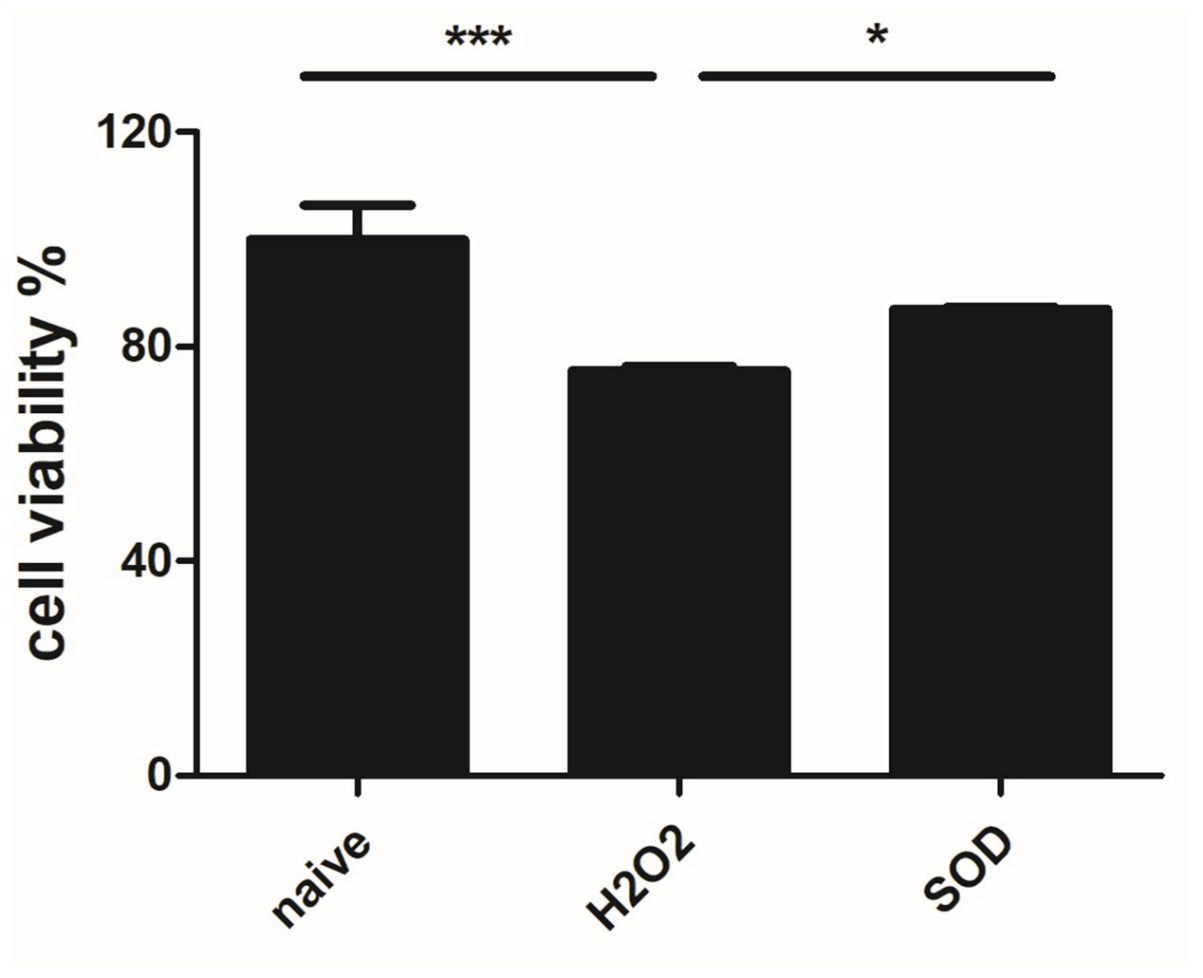
Effect of SOD on the viability of H_2_O_2_-treated SH-SY5Y cells. Cell viability was reduced in H_2_O_2_-treated SH-SY5Y cells. However, SOD pretreatment increased the viability of these cells. *P < 0.05 and ***P < 0.001.

**Fig 3:**
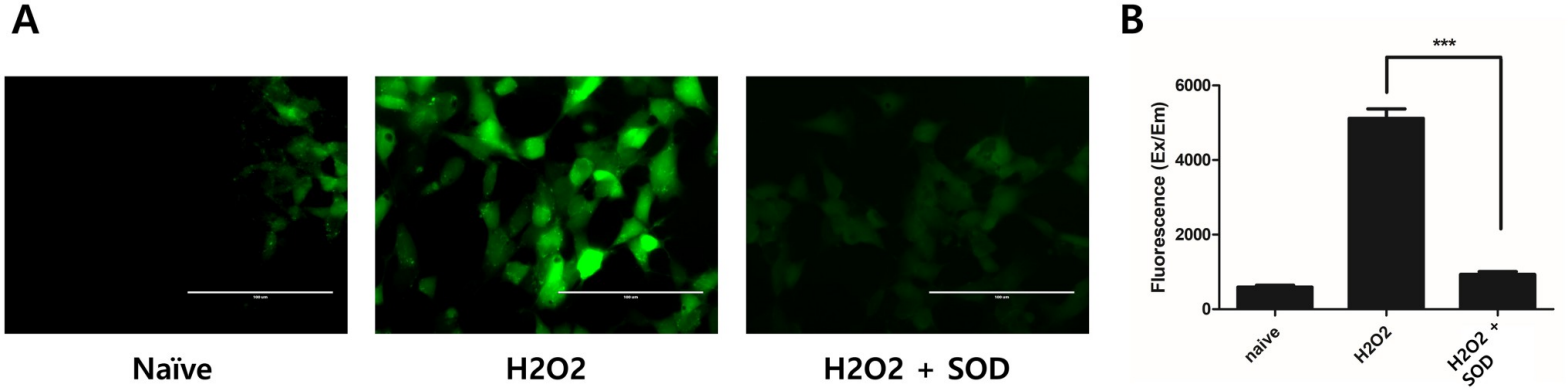
Effect of SOD on reactive oxygen species (ROS) accumulation. (A, B) H_2_O_2_ treatment increased ROS generation in SH-SY5Y cells, but this effect was diminished after the administration of SOD. ***P < 0.001.

SH-SY5Y neurons were first treated with H_2_O_2_ to induce oxidative damage. Thereafter, 1 μM SOD-pretreated and untreated SH-SY5Y cells were compared, and cell viability was evaluated using the CCK-8 assay. Cell viability was found to be increased in the SOD-treated group (*P* < 0.05; Fig 2). In addition, the degree of ROS relaxation was compared between the two groups using H_2_DCFDA and a fluorescent dye. ROS were expressed in H_2_O_2_-treated SH-SY5Y cells, and their expression levels were decreased after pretreatment with SOD (*P* < 0.001; Fig 3).

### Immunomodulatory effects of SOD on RAW264.7 cells

**Fig 4:**
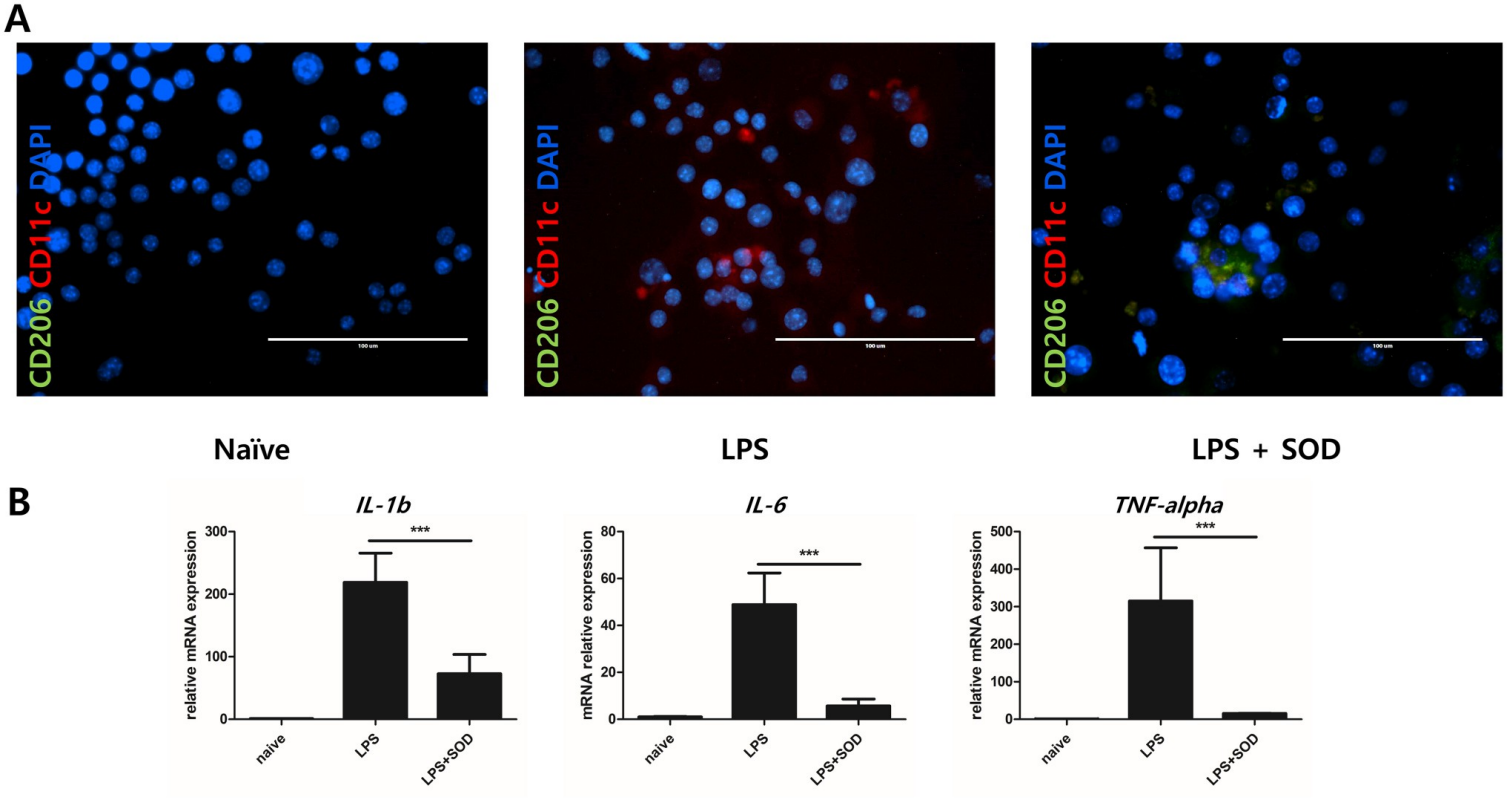
Change in macrophage polarization from M1 to M2 phase after SOD treatment. (A) Detection of CD11c (M1 marker, red) and CD206 (M2 marker, green) cells using an immunofluorescence assay. The proportion of CD11c cells was increased in the lipopolysaccharide (LPS)-treated group, whereas that of CD206 cells was increased in the SOD-treated cells. (B) mRNA expression levels of cytokines related to the M1 macrophage marker. The M1 phase was induced by LPS stimulation of macrophages and reversed by SOD treatment. The expression levels of interleukin (IL)-1β, IL-6, and tumor necrosis factor (TNF)-α were significantly increased in cells after stimulation with LPS. However, SOD treatment decreased the cytokine expression levels in these cells. ***P < 0.001.

The changes in macrophage polarization were observed after treating the RAW264.7 cells with SOD using IF staining with CD11c (red stain; marker of M1 phase) and CD206 (green stain; marker of M2 phases). CD11c expression, which is associated with M1 polarization, was increased in the LPS-treated macrophages, whereas CD206 expression, which is associated with M2 polarization, was increased in SOD-treated macrophages (Fig 4A).

Cytokine levels associated with the macrophage M1 phase, IL-1β, IL-6, and tumor necrosis factor (TNF)-α, were measured via qPCR. The RNA expression levels of IL-1β, IL-6, and TNF-α were increased in LPS-treated RAW264.7 cells but significantly decreased in SOD-treated RAW264.7 cells (*P* < 0.001; Fig 4B).

### SOD protects SH-SY5Y cells from the conditioned medium of RAW264.7 cells

**Fig 5:**
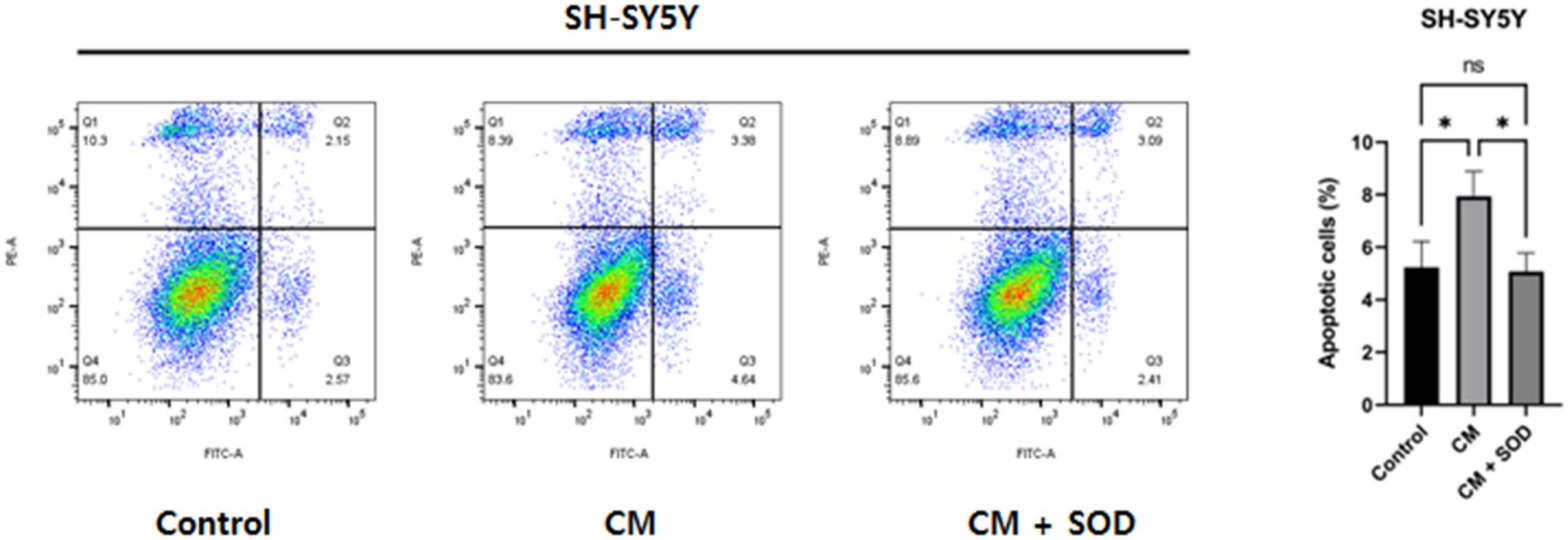
Reduced neuronal necrosis in SH-SY5Y cells treated with the conditioned medium after SOD treatment. Compared to the control group, neuronal necrosis was increased in the conditioned medium-treated group. In contrast, neuronal necrosis was decreased in the SOD-treated group compared to that in the SOD-untreated group. *P < 0.05. ns, not significant.

Conditioned medium from RAW264.7 cells was applied to SH-SY5Y cells. To confirm the neuroprotective effects of SOD, SH-SY5Y cells were treated with 1 μM SOD. Annexin V-FITC staining was performed to evaluate the necrosis of SH-SY5Y cells. Necrosis was increased in the nerve cells treated with the conditioned medium but decreased after SOD treatment (*P* < 0.05; Fig 5). These results indicate the neuroprotective effects of SOD.

## Discussion

SOD found in living cells prevents oxidative damage by regulating ROS levels. SOD is recognized not only for its antioxidant properties but also for its neuroprotective abilities [21]. SOD acts on various immune cells, such as lymphocytes and macrophages, to induce macrophage M2 polarization [22,23]. In this study, human neuroblastoma (SH-SY5Y) cells were used to evaluate the antioxidant and neuroprotective effects, whereas mouse macrophages (RAW264.7) were used to evaluate the immunomodulatory effects of SOD.

To determine the non-cytotoxic dose of SOD, a CCK-8 assay was performed. SOD was not toxic to macrophages at any concentration, but it decreased the cell viability above a certain threshold in nerve cells. Therefore, in this study, we determined the effects of 1 μM SOD, which is non-cytotoxic to both the selected cell lines, on nerve cells. To investigate the antioxidant effects of SOD, nerve cells were treated with H_2_O_2_ to induce oxidative damage, and ROS build-up was measured using a fluorescent product. ROS accumulation was increased in the H_2_O_2_-treated group, but it decreased significantly after SOD treatment. This indicates that SOD exerts antioxidant effects on SH-SY5Y cells. Subsequently, the cell viability was assessed. The decrease in cell viability in the presence of H_2_O_2_ was reversed after SOD administration. Notably, SOD not only exerted antioxidant effects by reducing ROS levels but also exerted protective effects on nerve cells, as established in previous studies [21,24].

SOD was applied to RAW264.7 mouse macrophages to assess its immunomodulatory capacity. Macrophages were treated with LPS to induce inflammation, and immunofluorescence staining and qPCR were performed to detect the release of inflammatory cytokines. IF staining revealed abundant red staining for CD11c (associated with M1 polarization) in LPS-treated macrophages and green staining for CD206 (associated with M2 polarization) in SOD-treated cells. qPCR revealed that the LPS-treated macrophages generated cytokines, such as IL-1β, IL-6, and TNF-α, which are associated with M1 polarization; however, SOD administration significantly reduced these cytokine levels (*P* < 0.001). These results indicate that SOD changes the phenotype of macrophages from M1 to M2.

LPS treatment of macrophages stimulates the shift of macrophages to the M1 phase as well as the release of cytokines associated with this phase. Therefore, conditioned medium derived from LPS-treated macrophages was used for nerve cells in this study. Treatment with the conditioned medium increased the necrosis of nerve cells relative to that in the control group; however, necrosis was decreased after the addition of SOD (*P* < 0.05). As the conditioned medium from LPS-stimulated macrophages is enriched with inflammatory cytokines, SOD may inhibit the functions of inflammatory cytokines in neural cells. SOD inhibits the production of inflammatory cytokines, such as IL-1β, IL-6, and TNF-α, in neutrophils [25]. In this study, SOD not only inhibited the production of macrophages but also protected the cells from inflammatory cytokines.

In this study, we confirmed the direct antioxidant and neuroprotective effects of SOD. Through an experiment with conditioned medium, the protective effects of nerve cells against inflammatory substances were confirmed. We also demonstrated the immunomodulatory effects of SOD and its ability to induce the polarization of macrophages from the M1 to M2 phase.

SOD acts as a potential therapeutic tool for several disorders [26], and ongoing studies are investigating its effects on neurodegenerative diseases. Neurodegenerative disorders, such as AD, Parkinson’s disease (PD), ALS, and multiple sclerosis, commonly exhibit microglia-mediated neuroinflammation [27]. Neuroinflammation requires microglial activation. In response to different microenvironmental disturbances, microglia can polarize into either the M1 pro-inflammatory or M2 anti-inflammatory phenotype [28]. Strategies maintaining the balance between microglial M1/M2 polarization have therapeutic potential for neurodegenerative disorders [29], and the microglial polarization shift from the M1 to M2 phenotype is a promising treatment target. Here, we showed that SOD stimulated macrophage polarization from the M1 to M2 phase. SOD supplementation reduced lipid peroxidation and maintained hippocampal neurogenesis to prevent cognitive decline in the stress-induced cells of mice [30].

In this study, SOD was toxic to nerve cells when its concentration exceeded a certain threshold. High doses of SOD decrease the myocardial contractility and trigger toxic reactions in post-ischemic heart models [31]. Therefore, determining the appropriate dosage is crucial for SOD treatment, with care taken to avoid overdosing.

Here, our experiments showed the antioxidant and neuroprotective effects of SOD on nerve cells as well as its ability to regulate the immune response of macrophages. Our findings suggest that SOD can be used to treat various diseases, particularly neurological diseases. As we only confirmed its effectiveness at the nerve cell level, future research is necessary to validate our findings in animal models. Furthermore, SOD toxicity at high concentrations should be thoroughly assessed in animal models.

## Conclusions

In this study, we demonstrated the antioxidant and neuroprotective effects of SOD on human nerve cells and its ability to control immunity in mouse macrophages. Our findings suggest SOD as a potential therapeutic agent for various diseases. This study demonstrated the efficacy of SOD at the nerve cell level; however, further animal research is necessary to validate our findings and facilitate the therapeutic application of SOD. Additionally, the protective mechanisms of SOD against inflammatory cytokines warrant further investigation.

## Abbreviations

AAMs: alternatively activated macrophages
CAMs: classically activated macrophages
CNS: central nervous system
H_2_O_2_: hydrogen peroxide
KO: knockout
LPS: lipopolysaccharide
ROS: reactive oxygen species
SOD: superoxide dismutase

## References

[1] Che M, Wang R, Li X, Wang HY, Zheng XFS. Expanding roles of superoxide dismutases in cell regulation and cancer. Drug Discov Today. 2016;21: 143–149. doi: 10.1016/j.drudis.2015.10.001 26475962 PMC4724522

[2] Altobelli GG, Van Noorden S, Balato A, Cimini V. Copper/zinc superoxide dismutase in human skin: Current knowledge. Front Med (Lausanne). 2020;7: 183. doi: 10.3389/fmed.2020.00183 32478084 PMC7235401

[3] Nguyen NH, Tran GB, Nguyen CT. Anti-oxidative effects of superoxide dismutase 3 on inflammatory diseases. J Mol Med (Berl). 2020;98: 59–69. doi: 10.1007/s00109-019-01845-2 31724066

[4] Corpas FJ, Fernández-Ocaña A, Carreras A, Valderrama R, Luque F, Esteban FJ, et al. The expression of different superoxide dismutase forms is cell-type dependent in olive (Olea europaea L.) leaves. Plant Cell Physiol. 2006;47: 984–994. doi: 10.1093/pcp/pcj071 16766574

[5] Kim SH, Kim SH, Lee JH, Lee BH, Yoon HJ, Shin DH, et al. Superoxide dismutase gene (SOD1, SOD2, and SOD3) polymorphisms and antituberculosis drug-induced Hepatitis. Allergy Asthma Immunol Res. 2015;7: 88–91. doi: 10.4168/aair.2015.7.1.88 25553268 PMC4274475

[6] Nojima Y, Ito K, Ono H, Nakazato T, Bono H, Yokoyama T, et al. Superoxide dismutases, SOD1 and SOD2, play a distinct role in the fat body during pupation in silkworm Bombyx mori. PLOS ONE. 2015;10: e0116007. doi: 10.1371/journal.pone.0116007 25714339 PMC4340916

[7] Fujii J, Homma T, Osaki T. Superoxide radicals in the execution of cell death. Antioxidants (Basel). 2022;11. doi: 10.3390/antiox11030501 35326151 PMC8944419

[8] Mittal M, Siddiqui MR, Tran K, Reddy SP, Malik AB. Reactive oxygen species in inflammation and tissue injury. Antioxid Redox Signal. 2014;20: 1126–1167. doi: 10.1089/ars.2012.5149 23991888 PMC3929010

[9] He L, He T, Farrar S, Ji L, Liu T, Ma X. Antioxidants maintain cellular redox homeostasis by elimination of reactive oxygen species. Cell Physiol Biochem. 2017;44: 532–553. doi: 10.1159/000485089 29145191

[10] Rosa AC, Corsi D, Cavi N, Bruni N, Dosio F. Superoxide dismutase Administration: A review of proposed human uses. Molecules. 2021;26. doi: 10.3390/molecules26071844 33805942 PMC8037464

[11] Sah SK, Agrahari G, Kim TY. Insights into superoxide dismutase 3 in regulating biological and functional properties of mesenchymal stem cells. Cell Biosci. 2020;10: 22. doi: 10.1186/s13578-020-00386-3 32128111 PMC7045732

[12] He C, Carter AB. The metabolic prospective and redox regulation of macrophage polarization. J Clin Cell Immunol. 2015;6. doi: 10.4172/2155-9899.1000371 26962470 PMC4780841

[13] Yao Y, Xu XH, Jin L. Macrophage polarization in physiological and pathological pregnancy. Front Immunol. 2019;10: 792. doi: 10.3389/fimmu.2019.00792 31037072 PMC6476302

[14] Orecchioni M, Ghosheh Y, Pramod AB, Ley K. Macrophage Polarization: Different Gene Signatures in M1(LPS+) vs. classically and M2(LPS-) vs. Alternatively Activated Macrophages. Front Immunol. 2019;10: 1084. doi: 10.3389/fimmu.2019.01084 31178859 PMC6543837

[15] Wang N, Liang H, Zen K. Molecular mechanisms that influence the macrophage m1-m2 polarization balance. Front Immunol. 2014;5: 614. doi: 10.3389/fimmu.2014.00614 25506346 PMC4246889

[16] Manni ML, Tomai LP, Norris CA, Thomas LM, Kelley EE, Salter RD, et al. Extracellular superoxide dismutase in macrophages augments bacterial killing by promoting phagocytosis. Am J Pathol. 2011;178: 2752–2759. doi: 10.1016/j.ajpath.2011.02.007 21641397 PMC3124355

[17] He C, Ryan AJ, Murthy S, Carter AB. Accelerated development of pulmonary fibrosis via Cu,Zn-superoxide dismutase-induced alternative activation of macrophages. J Biol Chem. 2013;288: 20745–20757. doi: 10.1074/jbc.M112.410720 23720777 PMC3711337

[18] Devanney NA, Stewart AN, Gensel JC. Microglia and macrophage metabolism in CNS injury and disease: The role of immunometabolism in neurodegeneration and neurotrauma. Exp Neurol. 2020;329: 113310. doi: 10.1016/j.expneurol.2020.113310 32289316 PMC7237336

[19] Munawara U, Catanzaro M, Xu W, Tan C, Hirokawa K, Bosco N, et al. Hyperactivation of monocytes and macrophages in MCI patients contributes to the progression of Alzheimer’s disease. Immun Ageing. 2021;18: 29. doi: 10.1186/s12979-021-00236-x 34154615 PMC8215492

[20] Mammana S, Fagone P, Cavalli E, Basile MS, Petralia MC, Nicoletti F, et al. The role of macrophages in neuroinflammatory and neurodegenerative pathways of Alzheimer’s disease, amyotrophic lateral sclerosis, and multiple sclerosis: Pathogenetic cellular effectors and potential therapeutic targets. Int J Mol Sci(3). 2018;19. doi: 10.3390/ijms19030831 29533975 PMC5877692

[21] Huang HF, Guo F, Cao YZ, Shi W, Xia Q. Neuroprotection by manganese superoxide dismutase (MnSOD) mimics: Antioxidant effect and oxidative stress regulation in acute experimental stroke. CNS Neurosci Ther. 2012;18: 811–818. doi: 10.1111/j.1755-5949.2012.00380.x 22934841 PMC6493411

[22] Agrahari G, Sah SK, Bang CH, Kim YH, Kim TY. Superoxide dismutase 3 controls the activation and differentiation of CD4+T cells. Front Immunol. 2021;12: 628117. doi: 10.3389/fimmu.2021.628117 33717151 PMC7947887

[23] Tan HY, Wang N, Li S, Hong M, Wang X, Feng Y. The reactive oxygen species in macrophage polarization: Reflecting its dual role in progression and treatment of human diseases. Oxid Med Cell Longev. 2016;2016: 2795090. doi: 10.1155/2016/2795090 27143992 PMC4837277

[24] Dohare P, Hyzinski-García MC, Vipani A, Bowens NH, Nalwalk JW, Feustel PJ, et al. The neuroprotective properties of the superoxide dismutase mimetic tempol correlate with its ability to reduce pathological glutamate release in a rodent model of stroke. Free Radic Biol Med. 2014;77: 168–182. doi: 10.1016/j.freeradbiomed.2014.08.029 25224033 PMC4258548

[25] Yasui K, Baba A. Therapeutic potential of superoxide dismutase (SOD) for resolution of inflammation. Inflamm Res. 2006;55: 359–363. doi: 10.1007/s00011-006-5195-y 17122956

[26] Younus H. Therapeutic potentials of superoxide dismutase. Int J Health Sci (Qassim). 2018;12: 88–93. 29896077 PMC5969776

[27] Appel SH, Beers DR, Henkel JS. T cell-microglial dialogue in Parkinson’s disease and amyotrophic lateral sclerosis: Are we listening? Trends Immunol. 2010;31: 7–17. doi: 10.1016/j.it.2009.09.003 19879804 PMC4126423

[28] Guo S, Wang H, Yin Y. Microglia polarization from M1 to M2 in neurodegenerative diseases. Front Aging Neurosci. 2022;14: 815347. doi: 10.3389/fnagi.2022.815347 35250543 PMC8888930

[29] Song GJ, Suk K. Pharmacological modulation of functional phenotypes of microglia in neurodegenerative diseases. Front Aging Neurosci. 2017;9: 139. doi: 10.3389/fnagi.2017.00139 28555105 PMC5430023

[30] Balendra V, Singh SK. Therapeutic potential of astaxanthin and superoxide dismutase in Alzheimer’s disease. Open Biol. 2021;11: 210013. doi: 10.1098/rsob.210013 34186009 PMC8241491

[31] Nelson SK, Bose SK, McCord JM. The toxicity of high-dose superoxide dismutase suggests that superoxide can both initiate and terminate lipid peroxidation in the reperfused heart. Free Radic Biol Med. 1994;16: 195–200. doi: 10.1016/0891-5849(94)90143-0 8005514

